# P-32. Risk Factors for Mortality with Coagulase-negative Staphylococcus Bacteremia in Cancer Patients

**DOI:** 10.1093/ofid/ofaf695.261

**Published:** 2026-01-11

**Authors:** Joseph Ragan, Theres Alexander, Alexandra Hanretty, Geena Kludjian, Madeline King, Carlo Foppiano Palacios

**Affiliations:** Cooper Medical School of Rowan University, Camden, New Jersey; Cooper Medical School of Rowan University, Camden, New Jersey; Cooper University Hospital and Cooper Medical School of Rowan University, Philadelphia, Pennsylvania; Cooper University Hospital and Cooper Medical School of Rowan University, Philadelphia, Pennsylvania; Cooper University Hospital, Camden, New Jersey; Cooper University Health Care, Camden, New Jersey

## Abstract

**Background:**

Coagulase-negative Staphylococcus species (CoNS) are commensal skin flora that frequently contaminate blood cultures and may not always be indicative of bloodstream infection (BSI). However, in specific high-risk populations, such as patients with malignancies, CoNS is the leading cause of BSI, and has previously been identified as an independent risk factor for mortality. The purpose of this study was to identify risk factors for true CoNS BSI and associated mortality.

**Table 1:**
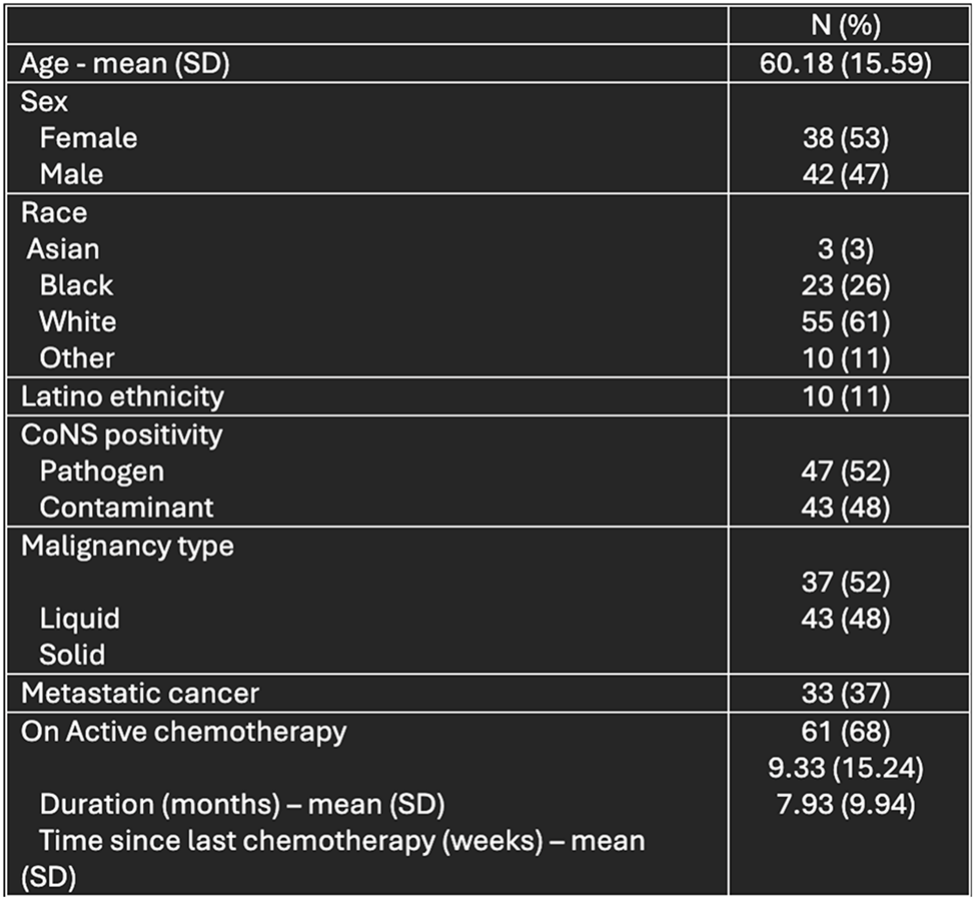
Baseline demographics

**Table 2:**
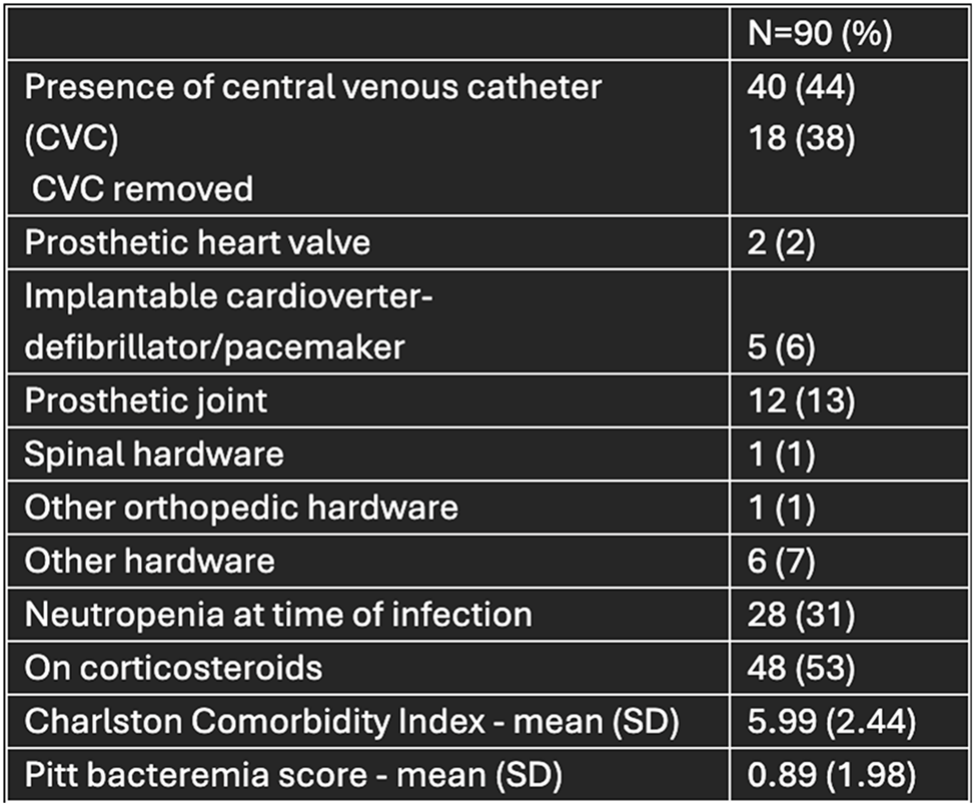
Patient features at time of infection

**Methods:**

This was a retrospective chart review of patients with solid and hematologic malignancy and positive CoNS blood culture from 2013-2023 at Cooper University Hospital. CoNS was deemed to be pathogenic or a contaminant based on chart documentation by infectious disease (ID) consultants or primary physician. Patients with CoNS deemed a contaminant represented the comparator group. Descriptive statistics, Fisher’s exact test, and Kruskal Wallis testing were performed.

**Table 3:**
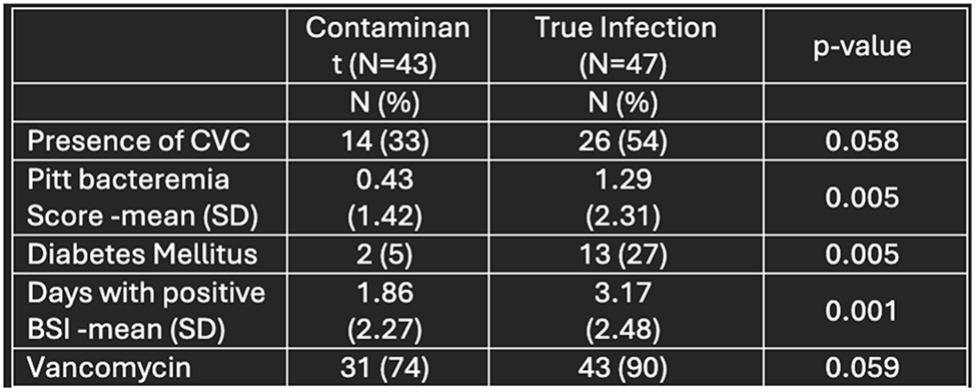
Factors associated with true BSI

**Table 4:**
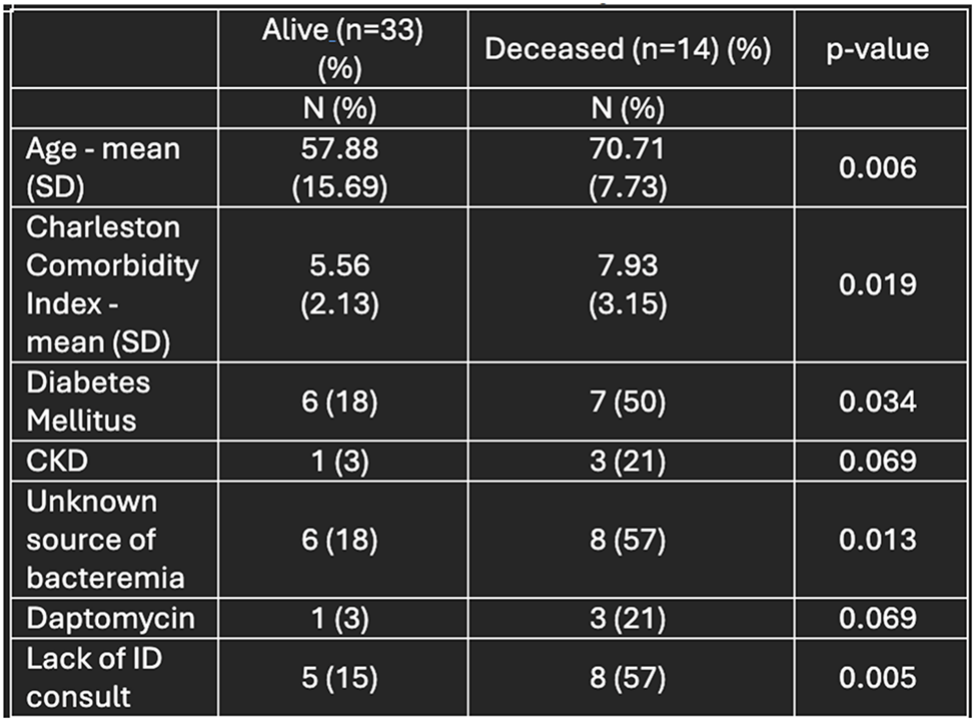
Factors associated with mortality in true infections

**Results:**

Ninety patients were included in the primary outcome analysis; 47 patients were identified as having a true BSI. Baseline demographics are in Table 1. Risk factors for true BSI included presence of CVC, Pitt bacteremia score, diabetes, increased number of days with positive blood culture, and the use of vancomycin. True BSI was associated with higher hospital mortality. Factors associated with mortality at discharge for true BSI included age, higher Charlson Comorbidity Index, diabetes, unknown source of bacteremia, use of daptomycin, and lack of ID consult.

**Conclusion:**

In cancer patients with blood cultures positive for CoNS, risk factors for true bacteremia included the presence of CVC and persistent bacteremia. This was further associated with a higher risk of in-hospital mortality. Interestingly, unknown source of infection and lack of ID consult were associated with higher mortality.

**Disclosures:**

Alexandra Hanretty, PharmD, Abbvie: Honoraria Madeline King, PharmD, Innoviva: Advisor/Consultant|Shionogi: Advisor/Consultant

